# The curative effect of Reduning injection combined with Xuanfeibaidu formula on COVID-19: A protocol for systematic review and meta-analysis

**DOI:** 10.1097/MD.0000000000022830

**Published:** 2020-11-13

**Authors:** Yunze Wang, Lizhu Han, Wei Zhang, Jing Sun

**Affiliations:** College of Pharmacy, Shaanxi University of Chinese Medicine, Xianyang, China.

**Keywords:** COVID-2019, meta-analysis, Reduning injection, Xuanfeibaidu formula

## Abstract

**Background:**

Since December 2019, COVID-19, caused by the new coronavirus (SARS-CoV-2), posed a serious threat to human health. On March 11, 2020, the World Health Organization announced that COVID-2019 has become a global pandemic. In China, Reduning injection (REDI) and Xuanfeibaidu formula (XFF) is widely used in treating COVID-19. However, there is no evidence-based medical evaluation that XFF combine with REDI is effective for COVID-19.

**Methods:**

The following databases will be searched: China National Knowledge Infrastructure (CNKI), Wanfang database, Chinese Science and Technology Periodical Database, Medline/PubMed, and Cochrane from October 1, 2019 to September 1, 2020. The suitable articles will be comprehensively and systematically searched without limitations of regions or language about REDI with XFF for COVID-19. The meta-analysis was performed by RevMan 5.3 and STATA 14.2 software.

**Results:**

This meta-analysis may help provide clarify on the effect of REDI combined with XFF to treat COVID-19. The result will be published at a peer-reviewed journal.

**Conclusions:**

This systematic review aims to provide new evidence of XFF combined with REDI for the treatment of COVID-19 in terms of its efficacy and safety.

**INPLASY registration number:**

INPLASY202090039.

## 1 Introduction

Since December 2019, a highly contagious coronavirus disease has spread the whole world.^[1]^ This novel virus belongs to *β*-coronavirus, which is highly similar to bat-SARS-coronavirus (bat-SL-CoVzc45). It is mainly transmitted by respiratory droplets and contact, and may also be transmitted by aerosol in a closed environment.^[2]^ The novel coronavirus disease (COVID-19), caused by infection of novel coronavirus, lead to pathological changes of lung, liver, spleen, kidney, and heart.^[3,4]^ As so far, COVID-19 has become a global pandemic attribute to this novel coronavirus is far more infectious than SARS.^[5,6]^ In a short period of 4 months, it has spread to more than 216 countries and regions, with a total of more than 27 million confirmed cases and more than 905, 426 deaths (as of September 11, 2020). For this harmful new pathogen, timely and accurate treatment is one of the important measures to determine individual infection status and control the further spread.^[7]^ During the outbreak of new coronavirus in China, traditional Chinese medicine (TCM) is widely used in treating COVID-19.^[8,9]^ Nevertheless, TCM treatment has the characteristic of “individuation”, so it is difficult to formulate standard treatment rules which leads to the uncertainty of clinical efficacy of TCM. Therefore, it is necessary to combine different types TCMs to improve the therapeutic effect.^[10]^ REDI, an important TCM injection, is used to treat COVID-19. It is mainly used for high fever, headache, cough, and other symptoms caused by upper respiratory infection (URI). In the eighth edition of COVID-19 diagnosis and treatment guideline issued by the China Health Commission, XFF is listed as one of the recommended medicine candidates. It inhibited virus infection and replication, the formula possessed anti-inflammatory effect and adjust the bodys immunological function after virus infection cells.^[11]^ According to this method, the review was updated to provide a strong evidence basis for clinical practice in the treatment of COVID-19. Therefore, we intend to collect randomized controlled trials (RCTs) about combining REDI with XXF for COVID-19 based on the basis of evidence-based medicine, and conduct a meta-analysis of its efficacy and safety.

## 2 Methods and analysis

### 2.1 Study registration

This systematic review was registered on INPLASY (INPLASY2020090039) on September 10, 2020. The DOI number is 10.37766/inplasy2020.9.0039. We strictly abide by Preferred Reporting Items for Systematic review and Meta-Analysis Protocols (PRISMA-P) guidelines.^[12]^

### 2.2 Inclusion and exclusion criteria

#### 2.2.1 Study design

In this study, all included studies were relevant randomized controlled trials (RCTs). Randomized studies can provide reliable clinical evidence, while non-randomize studies may lead to greater bias.

#### 2.2.2 Participants

Participants who clearly diagnosed with COVID-19 will be included in this study. There were no strict restrictions on gender and severity of the disease. According to “ Diagnosis and treatment of corona virus disease-19 (trial eighth editions) ” issued by the National Health Commission of China.^[13]^ The suspected cases have one of the following etiological or serological evidence:

1. Immediate fluorescent RT-PCR detection of novel coronavirus nucleic acid positive.
2. Detection of viral gene sequence infers that the sequence is highly homologous to known Novel coronavirus.
3. Characteristic IgM or IgG antibody of Novel coronavirus was displayed positive result.

#### 2.2.3 Intervention and comparator

The experimental group was treated with REDI combined with XFF to treat COVID-19, and the control group was treated with XFF alone. There were also no restrictions on the age, gender, or disease severity of the participants.

#### 2.2.4 Outcomes

Our primary outcomes were the disappearance rate of clinical features and the disappearance rate of mild symptoms.^[14]^ If other outcomes are reported in the eligible studies, these will be extracted and reported, but we will pay particular attention to the possibility of selective reporting bias when using any such outcomes in our review.^[15–18]^

### 2.3 Study search

**Figure 1:**
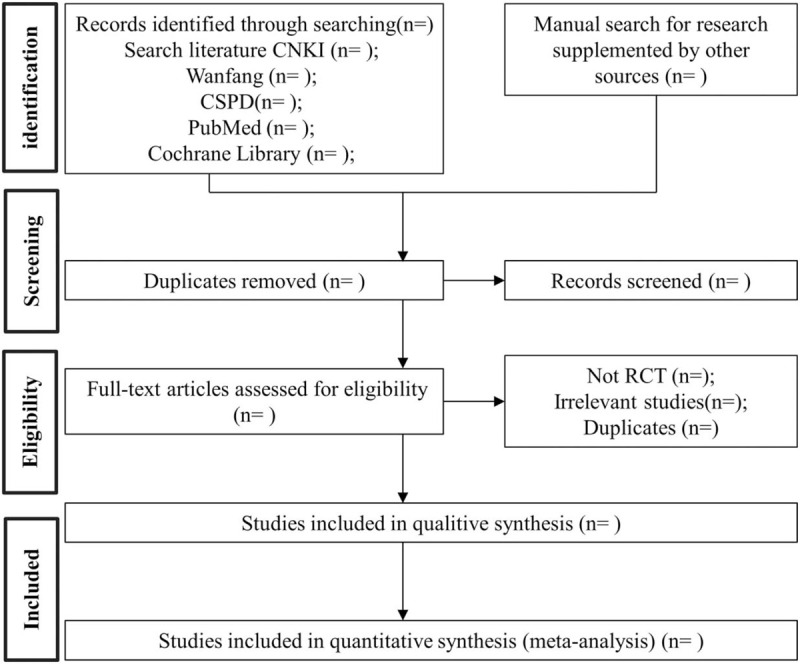
Flow diagram of study selection process.

Five databases including China National Knowledge Infrastructure (CNKI), Wanfang database, Chinese science and technology periodical database, Medline/PubMed, and the Cochrane Library were searched from December 1, 2019 to September 11, 2020. Among them, “meta-analysis”, “COVID-19”, “Reduning injection”, and “Xuanfeibaidu Formula” were used as keywords. The suitable articles will be comprehensively and systematically searched without limitations of regions or language about REDI with XXF for COVID-2019. Electronic database retrieval will be supplemented by manual retrieval of the included article reference list. The complete screening process is shown in Figure 1.

### 2.4 Study selection

EndNote X9, RevMan 5.3, and STATA 14.2 software will be used to screen the citations independently according to inclusion and exclusion criteria by 2 reviewers (Yunze Wang and Lizhu Han). If there were disagreements between 2 reviewers, a third reviewer (Wei Zhang) was available to check for accuracy. The above information was finally cross-checked by 2 reviewers.

### 2.5 Data extraction

According to the characteristics of the study, we prepare an excel table for data collection before data extraction. Outcome indicators of eligible studies were independently extracted and filled the outcomes in the data extraction table by 2 reviewers.^[19]^ The following data will be extracted: title, author details, participant characteristics, outcomes, and adverse events.^[20]^ To ensure the accuracy and consistency of the extracted data, cross-checked will be used by 2 reviewers.

### 2.6 Risk of bias assessment

We used the Cochrane bias risk and the symmetry of the funnel plot assessment to assess the methodological quality. The evaluation items include the following aspects: random sequence generation, assignment concealment, blinding method for patients, researchers and outcomes evaluators, incomplete outcome data, selective reports, and other sources of bias. Each of those items will be rated as high, low, or unclear risk of bias. We included all available studies to maximize the sample size and to enhance the generalizability of our findings.

### 2.7 Data analysis

The total effective rate, the main clinical feature disappearance rate, and the minor symptom disappearance rate were regarded as binary variables. Descriptive summary statistics in the form of mean, standard deviation, and range for continuous parametric measures were analyzed. Results will be calculated as mean difference (MD) and 95% CI. Dichotomous outcomes will be calculated with the odds ratio (OR) and 95% CI. Contents of inflammatory cytokines (IL-6, CRP, FER, TNF-α, WBC, IgG, IgM) are continuous variables, using weighted mean difference (WMD) or standardized mean difference (SMD) as the effect index, and 95% CI for description. The Q test was used for analysis, combined with *I*^2^ to quantitatively determine the degree of heterogeneity. If *P* ≥ 0.1 and *I*^2^ < 50%, it is considered that there is no statistical heterogeneity. We will examine the publication bias by evaluating the symmetry of the funnel plot. If the funnel plot is not symmetric, the results of the study may have a publication bias. If there is significant heterogeneity in our study, we will perform a subgroup analysis based on the type of control group. The experimental information in the study is incomplete, incomplete outcome data, and selective reports cannot judge the risk of bias. If there is significant heterogeneity in our study, we will perform a subgroup analysis based on the type of control group. Subgroup analyses by the statistical differences between the studies qualitative, critical illness rate, sample size, and quality score were performed.

## 3 Discussion

TCM has been widely used in the treatment of COVID-19, with satisfactory therapeutic effects and fewer side effects.^[21]^ During the prevalence of the novel coronavirus, the clinical efficacy data of the traditional Chinese medicine showed that combining different types TCMs has the effect of reducing patient mortality and improving the treatment effect of COVID-19.^[22]^ The XFF can reduce the infection rate of the novel coronavirus and play a preventive role. REDI can reduce the clinical symptoms of patients and has a good effect on the treatment. Clinical data shows that the 2 TCM have the effect of relieving the symptoms of lung inflammation and reducing the mortality of patients. Combining the 2 TCM can play a multi-target role, enhance the efficacy of the effect, and can also avoid unclear side effects that damage the patients body. Therefore, this study aimed to comprehensively evaluate the safety and the efficacy of REDI combined with XFF in COVID-19 patients through systematic reviews and meta-analysis. In other words, it can also provide new therapy for early control of COVID-19 and the novel coronavirus.

## Author contributions

**Conceptualization:** Yunze Wang.

**Data curation:** Lizhu Han.

**Funding acquisition:** Lizhu Han, Jing Sun.

**Investigation:** Wei Zhang.

**Methodology:** Wei Zhang.

**Software:** Wei Zhang.

**Supervision:** Jing Sun.

**Validation:** Jing Sun.

**Writing – original draft:** Yunze Wang.

**Writing – review & editing:** Jing Sun.
